# Patient-centred care and patient autonomy: doctors’ views in Chinese hospitals

**DOI:** 10.1186/s12910-022-00777-w

**Published:** 2022-04-08

**Authors:** Zhanming Liang, Min Xu, Guowei Liu, Yongli Zhou, Peter Howard

**Affiliations:** 1 The Second Affiliated Hospital of Shandong First Medical University, Taian, China; 2 James Cook University, Townsville, Australia; 3 Shandong University of Traditional Chinese Medicine, Jinan, China

**Keywords:** Chinese public hospitals, Doctor–patient relationships, Patient autonomy, Consumer participation, Health service quality

## Abstract

**Background:**

Patient-centred care and patient autonomy is one of the key factors to better quality of service provision, hence patient outcomes. It enables the development of patients’ trusts which is an important element to a better doctor-patient relationship. Given the increasing number of patient disputes and conflicts between patients and doctors in Chinese public hospital, it is timely to ensure patient-centred care is fully and successfully implemented. However, limited studies have examined the views and practice in different aspects of patient-centred care among doctors in the Chinese public hospitals.

**Methods:**

A quantitative approach was adopted by distributing paper-based questionnaires to doctors and patients in two hospitals (Level III and Level II) in Jinan, Shandong province, China.

**Results:**

In total, 614 doctors from the surgical and internal medicine units of the two hospitals participated in the survey yielding 90% response rates. The study confirmed the inconsistent views among doctors in terms of their perception and practice in various aspects patient-centred care and patient autonomy regardless of the hospital where they work (category II or category III), their unit speciality (surgical or non-surgical), their gender or seniority. The high proportion of doctors (more than 20%) who did not perceive the importance of patient consultation prior to determining diagnostic and treatment procedure is alarming. This in in part due to the belief held by more than half of the doctors that patients were unable to make rational decisions and their involvement in treatment planning process did not necessarily lead to better treatment outcomes.

**Conclusion:**

The study calls for the development of system level policy and organisation wide strategies in encouraging and enabling the practice of patient-centred care and patient autonomy with the purposes of improving the quality of the service provided to patients by Chinese hospitals.

**Supplementary Information:**

The online version contains supplementary material available at 10.1186/s12910-022-00777-w.

## Background

In medical ethics, patient autonomy in decision-making is demonstrated in the form of informed consent and the right of refusal in consideration of individual’s situation, constraints and capacities [1]. In healthcare settings, regardless of whether it is mandatary or optional, patient autonomy is centred around an individual’s responsibility to make health care decisions independently. The importance of patient autonomy is further reinforced by the introduction of patient ‘rights’ to information and informed choices such as alternative types of service, which has been witnessed in many healthcare systems globally such as in the UK National Health Service and the Australian public health system. Equity in access to health services and equality in health outcomes are two key measures for healthcare quality [2]. It is believed that these two quality elements cannot be met without individual patients being empowered and allowed to make decisions on treatment choices [3] and take moral responsibility for their choices and the use of resources represented by those choices [4].

However, like patient preferences, their competence in making autonomous decisions about service choices may need to be considered when developing system and organisational level policies in supporting patient autonomy. Given the nature of medical practice and the variation of patients’ knowledge of medical conditions and the complexity of treatment and prognosis, shared decision-making is more relevant and feasible. Under special circumstances, clinicians may be the best to act as patients’ agent in decision-making. However, patients’ welfare and wellbeing should be at the centre of the decision-making process which is enabled by adopting the patient-centred care (PPC) approach, an approach that can enhance the quality of care which impacts on patient outcomes and the patient–doctor relationship [5].

Although there is no universal definition of patient-centred care (PCC), the concept has been substantially advanced by the Picker Institute for the initiation of eight Picker Principles of Patient-Centred Care in 1993 [6]. ‘Respect for patients’ values, preferences and expressed needs’ and ‘Involvement of family and friends’ have been included as two out of the eight key PCC aspects.

There is a view that PCC is about a particular style that clinicians use to communicate and engage patients in active discussions [7] and empower patients to disclose preferences and contribute to decision-making processes [8]. This style contrasts with ‘doctor-centredness’ referring to a controlling and dominating gesture that doctors have upon patients in relation to diagnosis and treatment [9]. Studies have suggested that the most consistent elements of PCC are being attentive to patients’ physical and psychosocial needs and preferences; encouraging the disclosure of patients’ concerns and actively involving patients in decision-making [10, 11].

Fundamentally, patient-centred care requires a shift from the practice of authoritarian and domination of the clinician in the process of patient care provision to the acknowledgement of patient preferences and initiatives with a supportive and cooperative attitude [12–15]. Worth noting, patients’ preferences in relation to the degree of involvement in their care varies and can be influenced by many factors. There are circumstances that patients may prefer to solely rely on clinicians to make diagnostic and treatment decisions on their behalf [16], which should also be respected. It is feasible but challenging for clinicians to modify their participatory style to match patient preferences as it may not be an efficient way of anticipating patients’ expectations and identifying patient autonomy preferences without multiple consultations [17].

Trust is another key element that influences a patient’s satisfaction and patient-doctor relationships which, in turn, is a potential barrier to the practice of patient-centredness. Empirical evidence internationally and from China indicate that the erosion of trust is one of the major reasons behind medical disputes [18]. The medical profession earns patients’ trust and the promotion of autonomy not only from medical expertise and clinical effectiveness, but also by maintaining high standards of competence and moral responsibility, demonstrating respect, compassion, integrity and responsiveness to needs [19]. The basis of patient trust is the confidence that doctors will put their patients’ welfare ahead of all other considerations [22].

However, trust is a double-edged sword which not only refers to patients’ trust in doctors in terms of their professional expertise and desires in doing the best for them during the diagnosis and treatment process, but also doctors’ confidence in patients being rationale and respectful of their expertise and intention. Patients’ trust in clinicians is enhanced when clinicians are supportive of patients’ self-determination, giving patients a sense of autonomy, and try to understand their experiences and communicate clearly and honestly [21]. Such trust and patients’ perception of autonomy may foster satisfaction with physicians, which in turn, improves doctor-patient relationship leading to patient compliance of treatment and better patient outcomes [22].

The success of PCC cannot rely solely on clinicians’ efforts in changing the way they interact and communicate with patients; a comprehensive approach supported by organizational policy and strategies is needed which may include leadership support, a supportive work environment for clinical staff and systematic measure and feedback etc. [23].

The concepts of patient-centred care and patient autonomy are not new in the Chinese healthcare context. However, its focus has been more around informed consent, rather than taking patients’ preferences into account or allowing autonomous or shared-decision making or empowering patients to make rational decisions in health service choices. An increasing number of patient disputes and tensed doctor-patient relationships in Chinese public hospitals have drawn headlines during the past decade [24]. The incentive behind patient autonomy and shared decision-making is more about avoiding patient disputes rather than improving quality of patient care and patient outcomes [25]. Our search of literature published in the past 15 years has confirmed that only a handful of studies examining the practice of patient autonomy and shared decision-making in Chinese public hospitals have been published. Studies indicate the lack of patient participation in making service choices [25–27]. The reasons for doctors not actively encouraging shared decision making with their patients include time constraints, ineffective communication due to the lack of medical knowledge amongst patients [25], difficulties in assessing and understanding patients’ preferences [26] and inability to build the trust between doctors and patients which is critical to shared decision-making [26, 27].

Although recent publications in various Chinese journals have shown increasing interest in reinforcing the importance of patient autonomy and effective communications between doctors and patients for the benefits in improving patient satisfaction and service quality, no studies have been found specifically investigating doctors’ perceptions and practices in involving and consulting patients during the diagnostic and treatment process and whether such perceptions and practices are significantly influenced by the hospital context, the nature of medical practice and the seniority of the doctors’ position. In order to fill the missing knowledge gap, a large-scale study was conducted to examine the professional identity of the medical doctors and their practices during their initial patient encounter in the Chinese public hospital in late 2018 and early 2019. The purpose of the paper is to discuss some findings that may help develop an understanding of doctors’ perceptions and practices in consulting patients and involving patients in the process of making diagnostic and treatment decisions which is a core component of patient-centred care.

## Methods

A quantitative approach was adopted by sending paper-based questionnaires to potential participants. The surveys were conducted within four weeks in October 2019. Study participants were invited from one Level III—Qian FoShan hospital (QFSH), and one Level II hospital-LaiWu hospital (LWH), located in Jinan, the capital city of Shandong Province.  In the Chinese healthcare system, hospitals are categorised by level. Level III are the large teaching hospitals that provide more complex care with research and clinical teaching capacity. Level II hospitals are located in a suburb of large cities or in medium sized cities, and contain more than 100 beds, but less than 500. Level III are small community hospitals usually located in rural townships that contains less than 100 beds. The target population was medical doctors working in the medical inpatient units (n = 328) or the surgical inpatient units (n = 351). Eighty percent of all medical doctors from both hospitals working in the targeted units were invited to participate in the study. The survey took approximately 15 min to complete. A participant information sheet together with informed consent was given together with the survey questionnaire to all participants. Written consent was received from each of the participants.

### Questionnaires

The questionnaire was developed in English before translation into Mandarin Chinese. To maintain accuracy, it was then back translated into English by an independent collaborator and any adjustments were applied to the Chinese version. The questionnaire included 37 multiple choice questions to collect information on demographic, educational background and clinical position and ranking. Twelve out of the 37 questions focus on exploring doctors’ views/actions in relation to patient consultation and seeking patients’ preference and consent when planning diagnostic procedures and treatment. These 12 questions (Appendix) were devised on an understanding of the literature on what constitutes patient consultation or consultative approaches. Results of these 12 questions are included in the current paper. The draft survey was piloted with eight patients in both surgical and medical units before finalisation. Details of the questionnaire is provided as the Additional file 1. 

### Data analysis

Data from the two sets of the paper-based questionnaires were manually double entered into two MS Excel files and underwent error checking. The data were then imported into one IBM SPSS version 25 file. Descriptive statistics were performed on all variables separately by respondent. The dependent variables were then analysed by independent variables such as hospital, unit type, doctor seniority, gender by cross tabulation and chi square tests.

### Ethical considerations

The study received ethics clearance from La Trobe University Ethics Committee (HEC19251 date: 30 March 2019) and also received approval from the Research Committees of Qianfoshan Hospital and LaiWu Hospital for conducting the study with their doctors (Table 1).

**Table 1:** Target population and participation by hospital and unit

Unit	Hospital	Total target	Total participants	Response rate (%)
Surgical units	QFSH	308	279	91
	LWH	43	37	86
Medical units	QFSH	277	251	91
	LWH	51	47	92
Total		679	614	90

## Results

The study yielded high response rates of about 90% for doctors who received an invitation to participate as detailed in Table 2.

**Table 2:** Devoting adequate time to patients

Doctors	Strongly disagree (SD) %	Disagree (D) %	SD/D %	Neutral %	Agree (A) %	Strongly agree (SA) %	SA/A %
All	1.6	6.7	8.3	15.7	43.3	32.6	75.9
From QFSH	1.3	5.9	7.2	15.4	46.2	31.2	77.4
From LWH	3.6	11.9	15.9	17.9	25.0	41.7	66.7
From surgical units	2.6	5.8	8.4	14.4	42.5	34.8	77.3
From non-surgical units	0.7	7.7	8.4	17.2%	44.1	30.3	74.4
Male doctors	2.2	6.7	8.9	14.4	43.3	33.3	76.6
Female doctors	1.0	6.7	7.7	16.8	43.4	32.0	75.4
Chief physicians	2.5	5.9	8.4	12.7	37.3	41.5	78.8
Deputy chief physicians	0.0	6.6%	6.6	9.1	51.2	33.1	84.3
Attending physicians	2.8	5.6	8.4	20.7	44.1	26.8	70.9
Residents	1.1	8.0	9.1	17.1	41.7	32.1	73.8

The seniority of the study doctors was as follows:

- Chief physicians (19.4%),
- Deputy chief physicians (20.1%),
- Attending physicians (29.4%) and
- Residents (31.1%).

Twelve multiple choice questions included in the survey that were relevant to doctors’ views of patient autonomy and consultation are presented and discussed below. Responses to each of the questions from the following subgroups were compared to look for similarities and differences:

1. Doctors from QFSH versus doctors from LWH;
2. Doctors from surgical inpatient units versus doctors from internal medicine units
3. Male versus female doctors, and
4. Doctors of different seniority: chief physician, deputy chief physician, attending physician and resident.

For each question, results are provided to include percentage distribution of responses amongst all participants and by the subgroups for some questions when differences in the responses to specific questions existed amongst subgroups and the results of the appropriate statistical analysis to test the significance in difference is reported.

### **Question 1**

From your experience, patients’ improved knowledge of medicine requires doctors’ to improve their competence in making diagnosis and proving treatment?From your experience, patients’ improved knowledge of medicine requires doctors’ to improve their competence in making diagnosis and proving treatment?

The vast majority of the doctors (91.5%) agreed with the statement that patients’ improved knowledge of medicine requires doctors to improve their competence in making diagnosis and providing treatment. This view was consistent amongst participants without significant differences provided by subgroups.

### **Question 2**

 From your experience, patients make better treatment choices if they are involved in the planning process?

Close to 50% of all doctors agreed with the statement. However, 26.5% of all doctors disagreed that patient can receive better treatment outcomes if they were involved in the treatment planning process. Compared to the views of doctors from non-surgical units, doctors from surgical units were significantly more likely to agree with the statement. (58.7% versus 40.3%). Chi-Square = 27.70, df = 4, *p* < 0.0005. Compared to female doctors, male doctors were significantly more likely to agree that patients can receive better treatment outcomes if they are involved in the planning process (55.4% versus 44.0%). Chi-Square = 10.941, df = 4, p - 0.027. There are no significant differences in the views between other subgroups.

### **Question 3**

 From your experience, it is important for patients to be involved and consulted during the diagnosis and treatment process to achieve better outcomes.

Approximately 80% doctors agreed with the statement and close to 10% of them disagreed with the importance for patients to be involved and consulted during the diagnosis and treatment process to achieve better outcomes. Compared to doctors from non-surgical units, doctors from surgical units were significantly more likely to agree with the statement (84.7% versus 76.9%). Chi-Square = 15.132, df = 4, *p* = 0.004. There were no significant differences in the views between the other subgroups.

### **Question 4**

 From your experience, patients are not capable of making rational decisions regarding their health care needs.

Close to 60% of the participants agreed that patients are not capable of making rational decisions regarding their healthcare need. Compared to female doctors, male doctors were significantly more likely to agree that patients are not capable of making rational decisions regarding their health care (64.4% versus 53.0%). Chi-Square = 11.098, df = 4, *p* = 0.025. There are no significant differences in the views between other subgroups.

### **Question 5**

 From your experience, it is **not** necessary to consult patients about the types of diagnostic procedures that are required.

About 22% of the participants (24.4% from surgical unit and 19.3% from non-surgical unit) agreed that it is not necessary to consult patients about the types of diagnostic procedures that are required. Compared to male doctors, female doctors were significantly more likely to disagree with this statement (70.2% versus 59.1%). Chi-Square = 12.424, df = 4, *p* = 0.014. There were no significant differences in the views of other subgroups.

### **Question 6**

 Are you able to devote adequate time to each of your patients during the diagnostic and treatment process?

About 75.9% of the doctors agreed that they were able to devote adequate time to each of their patients during the diagnostic and treatment processes. Compared to doctors from LWH, doctors from QFSH were significantly more likely to agree that they were able to devote adequate time to each of their patients during the diagnostic and treatment processes. (77.4% versus 66.7%). Chi-Square = 18.353, df = 4, *p* = 0.003. Deputy chief physicians were significantly more likely to agree that they were able to devote adequate time to each of their patients during the diagnostic and treatment processes compared to other seniority levels (84.3% versus 77.8%). Chi-Square = 8.087, df = 3, *p* = 0.044. Details of the percentage distribution of responses amongst all participants and by the subgroups are included in Table 2.

### **Question 7**

 Doctors should make their own judgements without being influenced by patients’ preferences.

About 76% of the doctors agreed that they should make their own judgements without being influenced by patients’ preferences. There are no significant differences in the views between other subgroups.

### **Question 8**

 How often do you consult patients before determining the types of tests and procedures to be performed that assist with making diagnostic decisions?

Half of the doctors confirmed that they always consult patients before determining the types of tests and procedures to be performed that assist with making diagnostic decisions. Another 39% of the doctors said that they did consult patients 75% of the time. Less than 4% of doctors indicated that they rarely or did not consult patients for such purposes. There were no significant differences in the practice amongst subgroups.

### **Question 9**

 Choose one out of the five statements in relation to patients’ consent when determining the types of diagnostic tests required.

1. Patient consent is required for all procedures and tests
2. Patient consent is required for major procedures and tests
3. Patient consent is required for non-standard procedures and tests only
4. Patient consent to procedures and tests are only required when patient is required to make the decision
5. No patient consent to procedure and tests is necessary as doctors always have the final say

Sixty-two percent of doctors agreed that patients’ content is required before determining all or major procedures and tests. Three out of 614 doctors indicated that no patients consent to procedure and tests is necessary as doctors always have the final say. Compared to doctors from non-surgical units, doctors from surgical units were significantly more likely to agree that patients’ consent is needed for all diagnostic tests. (45.2% versus 27.9%). Chi-Square = 25.924, df = 4, *p* < 0.0005. Details of the percentage distribution of responses amongst all participants and by the subgroups are included in Table 3.

**Table 3:** Patients’ consent to diagnostic tests

Doctors	Answer 1 %	Answer 2 %	Answer 3 %	Answer 4 %	Answer 5 %
All doctors	**36.8**	**25.2**	23.9	13.7	0.5
From QFSH	37.9	24.6	23.1%	13.8%	0.6
From LWH	29.8	28.6	28.6%	13.1%	0.0
From surgical units	45.2	18.8	21.0	14.6	0.3
From non-surgical units	27.9	31.9	26.8	12.8	0.7
Male doctors	37.6	23.5	23.8	14.5	0.6
Female doctors	36.0	27.0	23.7	13.0	0.3
Chief physicians	30.8	32.5%	23.9	12.8	0.0
Deputy chief physicians	35.2	23.0%	29.5	11.5	0.8
Attending physicians	32.4	27.9%	22.3	17.3	0.0
Medical residents	46.0	19.6%	20.6	12.7	1.1

### **Question 10**

 Choose one out of the five statements in relation to patients’ consent when deciding their treatment plan.

1. Patient consent is needed for all treatment
2. Patient consent is needed for major treatment
3. Patient consent is needed for non-standard treatment only
4. Patient consent for treatment is only required when patient is required to make the decision
5. No patient consent for treatment is necessary as doctors always have the final say

Sixty-nine percent of doctors agreed that patients’ consent is required before determining all or major treatment for patients. Five out of 614 doctors (less than one percent) indicated that no patients’ consent to treatment is necessary as doctors always have the final say. Compared to doctors from non-surgical units, doctors from surgical units were significantly more likely to agree that patients’ consent is needed for all treatments. (43.9% versus 22.2%). Chi-Square = 37.058, df = 4, *p* < 0.0005. Details of the percentage distribution of responses amongst all participants and by the subgroups are included in Table 4.

**Table 4:** Patients’ consent to treatment plans

Doctors	Answer 1 %	Answer 2 %	Answer 3 %	Answer 4 %	Answer 5 %
All doctors	33.4	35.4	18.0	12.4	0.8
From QFSH	33.6	37.0	16.5	12.0	0.9
From LWH	32.1	25.0	27.4	15.5	0.0
From surgical units	43.9	32.2	12.1	11.1	0.6
From non-surgical units	22.2	38.7	24.2	13.8	1.0
Male doctors	31.8	39.2	16.4	11.6	1.0
Female doctors	35.1	31.4	19.4%	13.4	0.7
Chief physicians	29.3	42.2	19.0	9.5	0.0
Deputy chief physicians	32.8	36.1	21.3	8.2	1.6
Attending physicians	32.4	34.6	17.3	15.1	0.6
Medical residents	37.6	31.7	14.8	14.8	1.1

### **Question 11**

 How often do you face with ethical dilemmas in your work that are hard to resolve? (Percentage distribution)

As shown in Table 5, about 19.2% of all doctors confirmed that they very often or always had to face with ethical dilemmas during the care process. In contrast, 36.3% of all doctors indicated that they never or rarely had to face with ethical dilemmas. Compared to doctors from LWH, doctors from QFSH faced ethical dilemmas that were hard to resolve significantly more often (never: (20.7% versus 9.7%). Chi-Square = 16.409, df = 4, *p* = 0.003.

**Table 5:** Frequency of ethical dilemmas

Doctors	Never %	Rarely %	Never/ Rarely %	Sometimes %	Often %	Always %	Often/ Always %
All doctors	6.6	29.7	36.3	44.5	12.8	6.4	19.2
From QFSH	5.9	31.3	37.2	42.1	13.5	7.2	20.7
From LWH	11.0	19.5	30.5	59.8	8.5	1.2	9.7
From surgical units	4.1	31.8	35.9	43.9	11.5	8.6	20.1
From non-surgical units	9.2	27.5	36.7	45.1	14.2	4.1	18.3
Male doctors	4.2	24	29.0	46.8	14.8	9.4	24.2
Female doctors	8.7	34.9	43.6	42.3	10.7	3.4	14.1
Chief physicians	4.3	27.6	31.9	48.3	12.9	6.9%	19.8
Deputy chief physicians	9.9	33.9	43.8	35.5	12.4	8.3	20.7
Attending physicians	5.0	32.4	37.4	45.8	11.7	5.0	16.7
Medical residents	6.9	25.0	31.9	47.3	14.4	6.4	20.8

The following results are derived from comparing the column proportions. Values in the same row and subtable not sharing the same subscript are significantly different at *p* < 0.05 in the two-sided test of equality for column proportions. Cells with no subscript are not included in the test. Tests assume equal variances. Tests were adjusted for all pairwise comparisons within a row of each innermost subtable using the Bonferroni correction.

Compared to doctors from surgical units, doctors from non-surgical units were significantly more likely to never face ethical dilemmas that were hard to resolve. (67.5% versus 32.5%). In addition, compared to doctors from surgical units, doctors from non-surgical units were significantly less likely to always face ethical dilemmas that were hard to resolve. (30.8% versus 69.2%). Chi-Square = 12.637, df = 4, *p* = 0.013.

**Table:** 

‬‬‬‬‬‬‬‬‬‬‬‬‬‬‬‬‬‬‬‬‬‬‬‬‬‬‬‬‬‬‬‬	Never	Rarely	Sometimes	Often	Always
Surgical	32.5	55.2	50.9	46.2	69.2
Non-surgical	67.5	44.8	49.1	53.8	30.8

Compared to male doctors, female doctors were significantly more likely to never face ethical dilemmas that were hard to resolve. (66.7% versus 33.3%). In addition, compared to female doctors, male doctors were significantly more likely to always face ethical dilemmas that were hard to resolve (74.4% versus 25.6%). Chi-Square = 21.234, df = 4, *p* < 0.0005.

**Table:** 

‬‬‬‬‬‬‬‬‬‬‬‬‬‬‬‬‬‬‬‬‬‬‬‬‬‬‬‬‬‬‬‬	Never	Rarely	Sometimes	Often	Always
Male	33.3_a_	42.5_a, b_	53.5 _a, b_	59.0 _a, b_	74.4_b_
Female	66.7_a_	57.5 _a, b_	46.5 _a, b_	41.0 _a, b_	25.6_b_

### **Question 12**

 How often do you prescribe tests and procedures that are not necessary for patients, but for generating profit for the department and/or hospital?

Slightly more than 50% of the doctors have never prescribed tests and procedures that are not necessary to patients, but for generating profit for the department and/or hospital. However, between 10–20% of doctors indicated that they often or always did so. Compared to doctors from non-surgical units, doctors from surgical units were significantly more likely to prescribe unnecessary tests (often and always: 16.3% versus 9.8%). Chi-Square = 11.908, df = 4, *p* = 0.018. Compared to female doctors, male doctors were significantly more likely to prescribe unnecessary tests (often and always: 17.4% versus 8.7%). Chi-Square = 15.296, df = 4, *p* = 0.004.

For ease of understanding whether there are significant differences in the views held by different subgroups of doctors, Table 6 lists all questions that have found significant differences and types of differences.

**Table 6:** Significant differences in the distribution of responses amongst subgroups

Questions		Subgroup	Chi-Square	df	*p*
2	Patients can make better treatment choices if they are involved in the planning process	Units*	27.70	4	< 0.0005
		Gender**	10.941	4	0.027
3	It is important for patients to be involved and consulted during the diagnosis and treatment process to achieve better outcomes	Units*	15.132	4	0.004
4	Patients are not capable of making rational decisions regarding their health care needs	Gender**	11.098	4	0.025
5	It is not necessary to consult patients what types of diagnostic procedures are required	Gender***	12.424	4	0.014
6	Able to devote adequate time to each of your patients during the diagnostic and treatment processes	Hospital****	418.353		0.003
		Seniority*****	8.087,	3	0.044
9	Patients’ consent is required for all diagnostic procedures and tests	Units*	25.924	4	< 0.0005
10	Patients’ consent is required for all treatment	Units*	37.058	4	< 0.0005
11	Frequency of ethical dilemmas hard to resolve	Hospital***	16.409	4	0.003
		Units*	12.637	4	0.013
		Gender**	21.234	4	< 0.0005
12	Frequency of prescribing tests and procedures not necessary but for generating profit for the department and/or hospital	Unit*	11.908	4	0.018
		Gender***	15.296	4	0.004

* Doctors from surgical units are more likely to agree or strongly agree with the statement

** Male doctors are more likely to agree or strongly agree with the statement

*** Female doctors are more likely to agree or strongly agree with the statement

****Doctors at QFSH (Cat. III) are more likely to agree or strongly agree with the statement

*****Deputy Chief Physician are more likely to agree or strongly agree with the statement

## Discussion

The study achieved a high response rate from doctors from different seniority levels and from both surgical and non-surgical units working in the two targeted hospitals demonstrating a general interest in and commitment to the current topic. Chinese public hospitals and the medical profession recognise the urgency of improving the quality of hospital service provision as doctor-patient relationships have deteriorated significantly over the past decade for three major reasons; mistrust by patients and families, poor communications by medical professionals and system level barriers [18].

### Inconsistencies in the views and practices amongst doctors

In general, the study has confirmed inconsistencies in the views and practices amongst doctors regardless of the hospital where they work (category II or category III), their unit speciality (surgical or non-surgical), their gender or seniority. Responses to various questions indicate that the concepts of PCC and/or autonomy have not received full support from doctors and the variation in practices. More than a quarter of the doctors are making medical judgement without considering patients’ preferences and disagree that patients make better treatment choices if they are involved in the planning process, despite this PPC being a key element of practicing patient-centred care [6, 8]. This may be partially attributed by majority of the doctors’ belief that patients were not capable of making rational decisions regarding their health care needs. This is consistent with findings from recent studies conducted in China of the lack of involvement of patients in the care processes by clinicians [25–27].

The adequate time and attention devoted to patients during diagnostic and treatment process identified among more than a quarter of the doctors further confirmed negative attitudes of doctors toward patient involvement in the diagnostic and treatment process which would be addressed should PPC to be fully implemented in the public hospital system in China. Previous studies suggest that a lack of time and a poor understanding of patient expectations are two key barriers to the practice of patient-centred care [25–27].

The study indicates that doctors’ perceptions of the benefits of patient involvement and contribution to the treatment process may also play a part in the inconsistent practices and challenges the successful adoption of patient-centred care. More than half of the study participants did not believe in patients’ ability to make rational decisions and did not believe that better treatment outcomes would be achieved by involving them in the treatment planning process. This is a belief supported the traditional paternalistic approach to health care that positions the medical profession as the centre of an organisation around whom healthcare organisation’s workflow is based, and healthcare and its services are defined [12, 13]. This approach has been broadly criticised as it gives little consideration to patients’ preferences and tolerates decision making primarily based on the opinions and preferences of medical professionals [13] which compromises patients’ right to autonomy and shared decision-making. This belief opposes the foundation of patient-centred care. As a large proportion of doctors hold that opinion, to allow the successful implementation of patient-centred care, system and organisational level actions are required to allow the changes in current and future medical workforce rather than relying solely on doctor’s self-awareness and initiation of improvement.

Having said that, the literature acknowledges that an individual’s competence in decision-making and self-identification of personal values and preferences do impact on their ability to participate in shared decision-making [14, 15] and also in the way how they communicate with their clinicians. Evidence suggests that decision-making could be bias and not necessarily rational or logical and could be bias during ill health [29]. Encouraging patient autonomy could be a complex task for doctors who are required not to limit patients’ freedom of choices but at the same time helping individuals to make decisions that maximise their welfare. It is also important for doctors to provide comprehensive and objective information assist patient with weighing and evaluating the benefits and risks between choices, hence making informed decisions [30].

Good communications are not only built on the skills that medical professionals have developed at university or on the job training but is also heavily based on the respect and trust the patients and medical professionals have for each other [28]. However, an average increase of about 23% in the number of medical disputes in China since the early 2000’s [31] indicates the erosion of that trust between patients and doctors [18]. This can be attributed to reasons that can be addressed through training and professional development such as developing better communication and interpersonal skills, or reasons that are organisation-based such as heavy workload which may not be easily addressed, and reasons that are requiring systematic changes backed up by policies.

### Conflict of interests

It is believed that the volume-based incentive funding schemes adopted to fund public hospitals in China has encouraged revenue generating behaviours amongst doctors violating the trust and respect from patients and the public [32]. The current study confirms that only half of the doctors making diagnostics procedures and treatment choices for patients without taking ‘profit making’ into consideration. It is alarming to confirm that prescribing tests and procedures that are not necessary to patients is a common practice amongst 10–20% of doctors disregard their seniority, gender and hospital in which they are employed.

When doctors do not put the welfare of their patients ahead of other considerations, they fail to demonstrate their integrity, moral responsibility and compassion to patients resulting in a loss of patients’ trust [19]. This is one of the key attributing factors to increasing medical disputes and malpractice claims in China, which could be avoided should the doctor/patient relationships be improved [21]. When hospitals focus on avoiding patient disputes by settling/compensating malpractice claims and medical disputes privately without thorough investigation, they fail to protect their employees and create a culture that encourages ‘defensive medicine’ amongst doctors such as prescribing excessive diagnostic tests and medical procedures, and medication [33, 34]. Doctors’ fear of receiving complaints from patients and increasing doubt of patients’ ability in making rational decisions further damages the mutual trust between doctors and patients [21, 22].

Relevant to defensive medicine, sometimes doctors do face ethical dilemma at work that it is hard to resolve. In the current study, close to 20% of doctors indicate that they often face with ethical dilemmas in their work that are hard to resolve. Doctors from QFSH (20.7%) are significantly more likely to face the unresolved ethical dilemmas than doctors from LWH (9.7%). Female doctors and doctors working at the non-surgical units are significantly more likely to never had to face such difficult situation. The current study did not specify what are ‘ethical dilemma’ that doctors were facing, but literature does indicate one of which is allowing patients to accessing medical records and notes in written form as information provided exclusively verbally is still a common practice [35]. Although it was suggested that maximising information sharing including the access of patient records is an important step of patient-centred care and could improve patient trusts and increase patients’ sense of responsibilities [36], the questions of how much to be shared, when to share, and in what way it is shared are critical [37]. Patients and doctors’ views on information sharing are often conflicting, the pro and cons of sharing medical records and notes in real time requiring further evaluation to ensuring sharing information leads to better decision-making but not misinterpretation and unnecessary anxiety among patients [38].

### Differences between subgroups

The distribution of the responses was significantly different between various subgroups of doctors. The most frequent predictors of difference being unit type and gender. Hospital and seniority categories had limited effects on the views and practices amongst doctors in areas of patient consultation, consent and involvement. When doctors do not put the welfare of their patients ahead of other considerations, they fail to demonstrate their integrity, moral responsibility and compassion to patients resulting in a loss of patients’ trust [19]. This is one of the key attributing factors to increasing medical disputes and malpractice claims in China, which could be avoided should the doctor/patient relationships be improved [21]. When hospitals focus on avoiding patient disputes by settling/compensating malpractice claims and medical disputes privately without thorough investigation, they fail to protect their employees and create a culture that encourages ‘defensive medicine’ amongst doctors such as prescribing excessive diagnostic tests and medical procedures, and medication [33, 34]. Doctors’ fear of receiving complaints from patients and increasing doubt of patients’ ability in making rational decisions further damages the mutual trust between doctors and patients [21, 22].

The study clearly calls for systematic support and guidance to be provided in order to fully and successfully implement patient-centred care in Chinese public hospitals. System level policy incentivise hospitals in developing clear procedure and protocols in guiding the execution of patient-centred care among clinicians with clear standard and code of practices [39]. A culture of patient-centred care may not be successful without the full understanding of the rights and responsibilities of patients and their families and associating boundaries [23]. Appropriate use of community advocacy, promotion and education may help building such understanding and improving the trusts between patients and clinicians, which ultimately improving patient experiences of care, quality of care, and patient outcomes [23, 27].

### Study strengths and limitations

The main strength of the study was its high participant response rate resulting in views and practices representative of the target population. The main limitation was the nature of the questions and responses, being based on the opinions of the participants with no confirmatory evidence. In turn, this may partially explain the inconsistencies across the range of responses. In addition, the survey instrument was developed based on various questions from studies conducted in other countries which may require prior validation.

## Conclusion

Patient-centred care and patient autonomy is a practice that can build trust and improve doctor-patient relationship, but these strategies are practiced inconsistently by doctors in Chinese public hospitals. Such practice may include consulting and involving patients in the diagnostic and treatment planning process, taking patients’ preferences into consideration, informed consent, and requiring both the commitment from clinicians and recognition, support and guidance from the hospitals. The study concludes that to switch from medical domination and a provider-focus to an emphasis on patient-centred care, will be critical to initiate changes to system-level policies, organisation-based strategies and the mindsets of the medical profession.

### Supplementary Information

**Additional file 1**. Doctors’ survey.

## Data Availability

The datasets used and/or analysed during the current study are available from the corresponding author on reasonable request.

## Appendix: Twelve survey questions

**Table:**

No	Question
1	From your experience, patients’ improved knowledge of medicine requires doctors’ to improve their competence in making diagnosis and proving treatment?
2	From your experience, patients make better treatment choices if they are involved in the planning process?
3	From your experience, it is important for patients to be involved and consulted during the diagnosis and treatment process to achieve better outcomes
4	From your experience, patients are not capable of making rational decisions regarding their health care needs
5	From your experience, it is not necessary to consult patients about the types of diagnostic procedures that are required
6	Are you able to devote adequate time to each of your patients during the diagnostic and treatment process?
7	Doctors should make their own judgements without being influenced by patients’ preferences
8	How often do you consult patients before determining the types of tests and procedures to be performed that assist with making diagnostic decisions?
9	Choose one out of the five statements in relation to patients’ consent when determining the types of diagnostic tests required Patient consent is required for all procedures and tests Patient consent is required for major procedures and tests Patient consent is required for non-standard procedures and tests only Patient consent to procedures and tests are only required when patient is required to make the decision No patient consent to procedure and tests is necessary as doctors always have the final say
10	Choose one out of the five statements in relation to patients’ consent when deciding their treatment plans Patient consent is needed for all treatment Patient consent is needed for major treatment Patient consent is needed for non-standard treatment only Patient consent for treatment is only required when patient is required to make the decision No patient consent for treatment is necessary as doctors always have the final say
11	How often do you face with ethical dilemmas in your work that are hard to resolve? (Percentage distribution)
12	How often do you prescribe tests and procedures that are not necessary for patients, but for generating profit for the department and/or hospital?

## Abbreviations

PCC: Patient-Centred Care
QFSH: Qian FoShan hospital
LWH: LaiWu hospital
