# Supplementary material for: Patient-centred care and patient autonomy: doctors’ views in Chinese hospitals
Source: BMC Med Ethics. 2022 Apr 8;23:38. doi: 10.1186/s12910-022-00777-w (PMC8994393; doi:10.1186/s12910-022-00777-w)
Supplement: Supplementary file 1 — Additional file 1. Doctors’ survey. [file 12910_2022_777_MOESM1_ESM.pdf]

Doctors' survey
医生调查问卷

1. Please select the classified level of the hospital where you are currently employed
   1. Category III
   2. Category II
   3. Category I

2．Please select the department that you are currently working in
   1. Non-surgical Unit
   2. Surgical Unit

3．What is the level of seniority of your medical position?
   1) Chief Physician
   2) Associate Chief Physician
   3) Attending Physician
   4) Resident

4. Have many years have you been working as a clinician in the hospital system (including hospitals that you previous worked currently working for)?

5. How many years have you been employed by the current hospital?

6. What is your age?

7. What is your gender?
   1) Male
   2)
   3) Female

8. Do you have any of the following degrees?
   1) TAFE
   2) Bachelors
   3) Master
   4) Doctorate or PhD

9. Please select the discipline(s) of all of the degrees that you obtained
   1) Medicine
   2) Nursing
   3) Management
   4) Others (please key in details)

All of the below questions are provided the same 5-point Likert Scale for selection of answer

1) Strongly disagree
2) Disagree
3) Either agree or disagree
4) Agree
5) Strongly agree

10. From your experience, patients' improved knowledge of medicine has pressured doctors' to be more rigorous in diagnosis and treatment?

11. From your experience, patient can receive better treatment outcome if they are involved in the treatment planning process?

12. From your experience, it is important for patients to be involved and consulted during the diagnosis and treatment process in order to receiving better treatment outcomes?

13. From your experience, patients are not capable of making rational decisions regarding their health care

14. From your experience, it is not necessary to consult patients the type of assisted diagnostic procedure

15. You are able to devote adequate time to each of your patient during the diagnostic and treatment process

16. There are sufficient rules to guide you when making decisions in the face of ethical dilemmas

17. Doctors should make their own judgement rather than relying on rules

18. Doctors should make their own judgement without concerning patients' preference

19. From your experience, patients demonstrate high level of respect to their doctors

20. In the past, you had concerns about your own safety when treating patients in critical condition

21. Did you feel well supported by your superior or hospital when there were disputes between you and your patients

22. Do you have confidence that you will be well supported by your superior or hospital when there are disputes between you and your patients?

All of the below questions are provided the same 5-point Likert Scale for selection of answer

1) Never
2) Rarely
3) Sometimes

4) Often
5) Always

23. How often do you consult patients before determining the types of test and procedures to be performed that can assist with making diagnosis decision?

24. How often do you consider ethical implication in your clinical decision making?

25. How often do you need to use plain language to explain diagnostic and treatment procedure to patients rather than using medical terminology?

26. How often are you able to reduce resistance from patients when asking questions that are confidential and private in nature?

27. How often do you prescribe tests and procedures that are not necessary to patients, but for generating profit for the department and/or hospital?

28. How often do you face with ethical dilemmas in your work that are hard to resolve?

29. How often do you encounter patients recording the diagnostic and treatment process by audio or video without your consent?

30. How often do you terminate the treatment if you encounter the above situation as in question 29?

31. How often were you able to complete the examination process when you encountered resistance from patients when performing physical examination on private body part?

Multiple choice, one answer only

32. The guiding philosophy of your being a doctor is
    1) to care for patient as a person including his/her physical and psychological well-being
    2) to preserve or restore patients' physical health
    3) to serve the community
    4) to provide services on demand

33. Do you agree with which of the following statement in relation to patients' consent on tests?
    1) patients' consent is needed in all procedures, tests and treatments
    2) patients' consent is needed in major procedures, tests and treatment
    3) patients' consent is needed in non-standard procedures, tests and treatment only
    4) Patients' consents to procedures, tests and treatments are only required when patient is required to make the decision
    5) no patients' consent to procedure, tests and treatment is necessary as doctors should always have the final say

34. Do you agree with which of the following statement in relation to patients' consent on treatment?

1) patients' consent is needed in all procedures, tests and treatments
2) patients' consent is needed in major procedures, tests and treatment
3) patients' consent is needed in non-standard procedures, tests and treatment only
4) Patients' consents to procedures, tests and treatments are only required when patient is required to make the decision
5) no patients' consent to procedure, tests and treatment is necessary as doctors should always have the final say

35. With whom do you think it is appropriate to discuss a patient's case first when patients are in critical condition?

1) Patients' themselves
2) Immediate family members
3) Patient's relatives
4) Other doctors
5) Superior
6) Others

36. How many of your patients can you recognise by face?

1) More than 75%
2) 50 – 74%
3) 25-49%
4) Less than 25%
5) None

37. From your experience, what are the primary reasons that lead to dispute between doctors and patients

1) Patients and/or carers' mistrust
2) Patients unrealistic expectation of treatment outcomes and prognosis
3) Patients experienced complication as a result of lack of compliance to medical order
4) Complexity of hospital procedure and protocol leading to the inconvenience to patients and/or carers
5) Necessary treatment and procedures are unaffordable to patients
6) Long waiting for consultation/procedure/treatment
7) Patients suffered from preventable adverse outcomes
8) Patients suffered from unavoidable adverse outcomes under the current medical knowledge and treatment capacity
9) Ineffective communication between patients and doctors
